# Pictorial adaptation of the quality of recovery 15 scale and psychometric validation into a pediatric surgical population

**DOI:** 10.1038/s41598-023-40673-w

**Published:** 2023-08-28

**Authors:** Eric Noll, Vincent De Angelis, Claire Bopp, Chloe Chauvin, Isabelle Talon, Elliott Bennett-Guerrero, François Lefebvre, Julien Pottecher

**Affiliations:** 1 Department of Anesthesiology, Critical Care and Perioperative Medicine, Hautepierre Hospital, Strasbourg University Hospital, Strasbourg, France; 2 Physiology Laboratory, Faculté de Médecine, Equipe Acceuil 3072, Strasbourg, France; 3 Department of Pediatric Surgery, Hautepierre Hospital, Strasbourg University Hospital, Strasbourg, France; 4 Department of Anesthesiology, Stony-Brook Medicine, Stony-Brook, NY USA; 5 Department of Biostatistics, Strasbourg University Hospital, Strasbourg, France

**Keywords:** Medical research, Paediatric research

## Abstract

Patient reported outcomes measures (PROMS) are important endpoints to measure patient health status in the perioperative setting. However, there are no good tools to measure PROMS in the pediatric surgical population. Patients 7 to 17 years old undergoing surgery were included and followed up for 1 day after surgery (POD1). At POD1 the patients were asked to rate their overall postoperative recovery using a 100-mm visual analog scale (VAS). The primary outcome was the pediatric QoR-15 score on postoperative day 1 (POD1). 150 patients completed the study. The mean (SD) pediatric QoR-15F scores were 132.1 (14.1) and 111.0 (27.0), preoperatively and on POD1, respectively. Convergent validity confirmed with Pearson (r) correlation between the postoperative pediatric QoR-15F and the patient-rated global recovery assessment was 0.72 (95% confidence interval [0.63–0.79]; p < 10^–16^). Concerning reliability, internal consistency of the pediatric QoR-15 assessed by Cronbach’s alpha was 0.90. The test–retest concordance correlation coefficient was 0.92; 95% CI [0.83–0.96]. Split-half alpha was 0.74. The pictorial pediatric version of the QoR-15F showed good validity, reliability, responsiveness, acceptability and feasibility. This PROMS should be considered for clinical care and research in the perioperative pediatric patient setting.

**Trial Registration:** NCT04453410 on clinicaltrials.gov.

## Introduction

It is estimated that approximately 4 million children undergo surgery in the United States annually^1^. Measuring quality of recovery after surgery is important for patients, clinicians and health systems^2–5^. The patient’s perspective concerning his/her health status is a validated endpoint to assess quality of recovery^6^. Questionnaires have been psychometrically validated to measure postoperative quality of recovery from the patient’s perspective^7,8^. Among these patient-reported outcomes measures (PROMs), the Quality of Recovery 15 (QoR-15) questionnaire, is one of the most extensively studied, translated and externally validated tools^9–14^.

This questionnaire, however, has not been yet adapted and validated for pediatric patients. Pictorial scales are an attractive solution to assess a child’s perspective^15,16^, e.g. for symptoms like pain or postoperative nausea^17,18^. Instruments for PROMs should be validated in every clinical setting, and the pediatric population may differ significantly from the adults in term of communication and behavioral characteristics.

This study was therefore designed to validate the psychometrical properties of an adapted version of the QoR-15 i.e., the pediatric QoR-15. We hypothesized that after pediatric-centered adaptations and replacement of the numbering rating scale with a pictorial scale, the pediatric QoR-15 would offer suitable performance to assess quality of recovery in a pediatric surgical setting.

## Methods

### Study design and patients

This is a single center prospective cohort study. The study was performed at the Hautepierre Hospital, Strasbourg University Health System in France. The study was approved by the Institutional Review Board on June the 20th 2020 (Comité de Protection des Personnes Est IV n°20/47, Strasbourg University Hospital, chairperson Prof EA Sauleau, approval # IDRCB 2020-A00689-30). All experiments were performed in accordance with the relevant guidelines and regulations. Enrollment was performed from September 2020 until September 2021.

Patients 7 to 17 years old scheduled to undergo surgery in the department of pediatric surgery at the Hautepierre Hospital and covered by the French national health insurance, were considered for enrollment. Exclusion criteria included: preoperative cognitive or sensory disorder that might impair interview reliability, surgery potentially inducing severe postoperative cognitive or sensory disorder (e.g., intracranial surgery), inability to speak French, and inability to provide informed consent to the study.

Written informed consent was obtained from patients and parents. Before the first study interview, a research coordinator tested the patient’s ability to self-report symptom severity: a seriation test and a classification test involving several clinical vignettes were completed as previously described^19^. One test involved polygonal paper forms classification according to their surface and the other test involved picture classifications accordingly to pain intensity (eg cardiac auscultation, scratched knee, forearm cast accounting for wrist fracture). These tests, adapted from a previously described study, aimed at screening children with ability to self-report symptom severity. Only patients passing these tests underwent further study procedures.

### Pediatric QoR-15 construction

The pediatric QoR-15 scale was constructed based on the French version of the QoR-15 scale, validated in the adult population (QoR-15F)^13^. Changes included language adaptation and replacement of the numbering rating scale with a pictorial scale. Language adaptation consisted of replacing the personal pronouns adapted for children in the questions and reformulation of question 8 to focus on school and hobbies instead of work. The pictorial scale was designed by the study team. Six different faces with varying emotional states were drawn using PowerPoint® drawing tools (Microsoft Office, Microsoft, USA). These six faces were then printed in separate sheets of papers. Ten volunteers including two children (6 and 8 years old) were then asked to classify the faces from the unhappiest to the happiest face. Every volunteer ended up to the same order and classification, which was thus integrated in the pediatric QoR-15 as the scaling tool, each face corresponding to a 2 point increment from 0 to 10 (see Supplementary Fig. 1).

### Variables

The primary outcome was the pediatric QoR-15 score performed on postoperative day 1 (POD1). Covariables extracted from the patient’s medical record included preoperative characteristics (age, American Society of Anesthesiology Physical Status, type of surgery, preoperative pediatric QoR-15 score), intraoperative data (type of anesthesia, duration of surgery) and postoperative data (PACU and hospital length of stay). Extent of surgery was classified as minor, intermediate or major, based on expected stress response, inflammation and duration of surgery as previously described^8,13^.

On POD1, patients were asked to rate their overall postoperative recovery using a 100-mm visual analog scale (VAS). The duration of completing this form was also measured.

Outpatient were assessed through a phone call to their parents who received a paper form of the scale at the end of the preoperative visit. The presence of any complication (including cardiovascular, infectious, respiratory, or renal complication) on postoperative day 1 was also recorded.

To minimize measurement bias, all pediatric QoR-15 form completions were conducted by two trained research coordinators including coauthor VDA.

### Statistical methods

As correlation analysis preclude reliable power calculation, a sample size of 150 patients was selected based on previous QoR-15 validation studies in adult patients^8,13^. Statistical analyses were performed similar to previously published studies in the adult setting^13^. Date are reported as mean (standard deviation, SD), median (inter-quartile range, IQR), or number (percentage) as appropriate. Distribution of the data was analyzed with the Shapiro–Wilk test and with quantile–quantile diagrams. For categorical data, inferential analysis was performed with the chi2 or Fisher’s exact test, as appropriate. For continuous data, inferential analysis was performed using the Student’s or Mann–Whitney-Wilcoxon test, as appropriate. For multiple comparisons, analysis of variance or the Kruskal–Wallis test was considered. Correlation was calculated with Pearson correlation coefficient. All analysis were performed with R software (R foundation for Statistical Computing, version 4.0.2, Vienna, Austria). Statistical signification wad defined by a two-tailed P < 0.05.

The psychometric validation strategy was similar to previously published methods^8,13^. First, validity was tested based on convergent, construct and discriminant validity. Convergent validity consisted of testing the hypothesis that there is a positive correlation between the postoperative QoR-15F score and the global recovery assessment as measured by the VAS. Inter-item correlation also assessed convergent validity. Construct validity was tested by the hypothesis that there is a negative association between duration of surgery, type of surgery (minor, intermediate, or major), duration of PACU stay, and hospital length of stay on the one hand and pediatric QoR-15 score on the other hand. Discriminant validity was tested with the hypothesis that patients without a complication have higher postoperative QoR-15 scores compared to patients with complications.

Second, reliability was tested with internal consistency based on Cronbach’s alpha, split half reliability. Test–retest reliability was analyzed in a subset of 26 patients who were randomly selected to undergo 2 separate pediatric QoR-15F testing on POD-1. Third, responsiveness was assessed with Cohen size effect and standardized response mean.

Fourth, acceptability and feasibility were assessed with successful completion rate and time to complete the questionnaire. There was no computation for missing data. All statistical analyses were supervised by a senior statistician (co-author FL).

## Results

**Figure 1:**
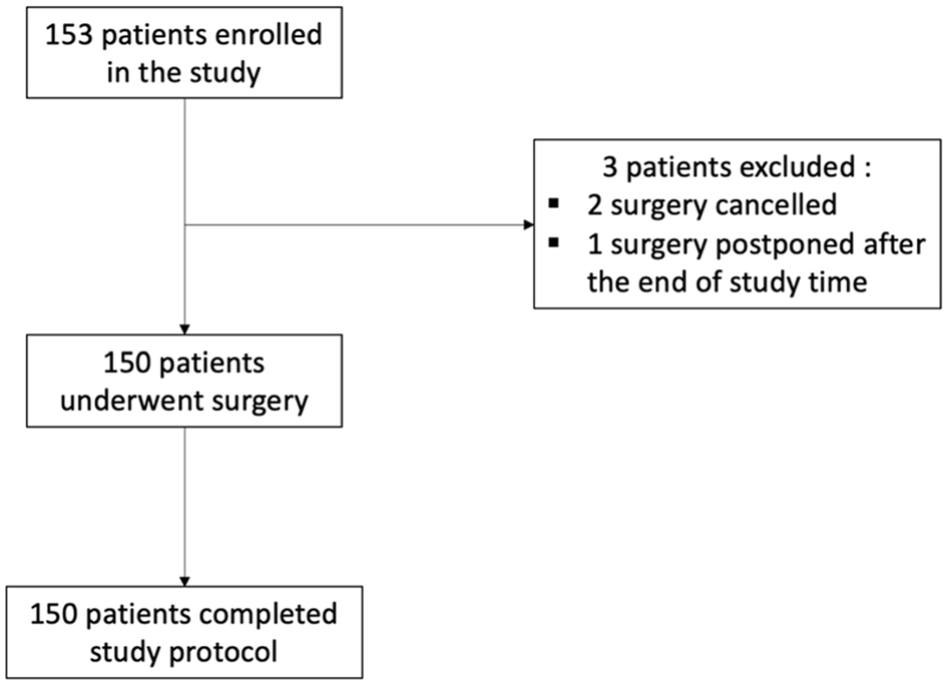
Study flow chart.

Overall, 153 patients were enrolled and 150 completed the study protocol (Fig. 1). Preoperative characteristics of the patients are presented in Table 1. The mean (SD) duration of surgery, PACU and hospital stay were 68 (69) min, 60 (41) min and 2.1 (2.4) days, respectively. Sixty-four percent of the patients underwent outpatient surgery. The mean (SD) preoperative and postoperative pediatric QoR-15F scores were 132(14) and 111 (27), respectively.

**Table 1:** Patient characteristics.

	n = 150
Age (years), mean (SD)	12.6 (2.9)
Weight, kg, mean (SD)	52 (19)
Height, cm, mean (SD)	155 (17)
Male sex n, (%)	78 (52%)
ASA physical status score n, (%)	
1	110 (73)
2	37 (25)
3	3 (2)
4	
Type of surgery	
Digestive	53 (35%)
Orthopaedic, spine	83 (55%)
Other	14 (9%)
Extent of surgery n, (%)	
Minor	74 (49)
Intermediate	45 (30)
Major	31 (21)
Type of Anesthesia n, (%)	
General	67 (45)
Regional	2 (1)
General and regional	81 (54)

*ASA* American society of Anesthesiology.

### Construct and convergent validity

Pearson (r) correlation between the postoperative pediatric QoR-15F and the patient-rated global recovery assessment was 0.72 (95% confidence interval (CI) [0.63–0.79]; p < 10^–16^). The pediatric postoperative QoR-15F was significantly lower in the group of patients rating their global recovery as poor (< 70 mm) compared to the patients rating their global recovery as good (≥ 70 mm) with these subgroups having a QoR-15 score of 98 (26) and 130 (15) respectively; p < 0.1 10^–13^. The mean pediatric Qor-15F in the minor, intermediate and major surgery groups were 124 (18), 104 (28) and 90 (27, respectively); p < 0.05). There was a negative correlation between postoperative pediatric QoR-15F on the one hand and duration of surgery (r = − 0.46 [− 0.57; − 0.32]), duration of PACU stay (r = − 0.37 [− 0.50; − 0.22]), and hospital length of stay (r = − 0.50 [− 0.61; − 0.37]) on the other hand. In the subgroups of patients without and with complications the pediatric mean QoR-15F scores were 112 (28) and 106 (28), respectively; p = 0.44. The inter-item correlation matrix is shown in Table 2.

**Table 2:** Inter-item correlation matrix for the Pediatric version of the French Quality of Recovery scale (pediatric QoR-15F) score on postoperative day 1.

Pediatric QoR-15F item	Correlation with pediatric QoR-15F item															Correlation with total QoR-15 score
	1	2	3	4	5	6	7	8	9	10	11	12	13	14	15	
1		**0.53**	**0.25**	**0.26**	**0.46**	**0.41**	0.11	**0.32**	**0.41**	**0.42**	**0.31**	**0.36**	**0.42**	**0.29**	**0.45**	**0.58**
2			**0.51**	**0.43**	**0.59**	**0.56**	0.10	**0.50**	**0.46**	**0.51**	**0.25**	**0.39**	**0.42**	**0.24**	**0.27**	**0.69**
3				**0.66**	**0.41**	**0.45**	0.12	**0.49**	**0.48**	**0.55**	**0.29**	**0.44**	**0.42**	**0.39**	**0.25**	**0.69**
4					**0.44**	**0.30**	0.09	**0.52**	**0.56**	**0.60**	**0.40**	**0.54**	**0.47**	**0.44**	**0.27**	**0.73**
5						**0.33**	0.02	**0.50**	**0.52**	**0.45**	**0.28**	**0.49**	**0.36**	**0.29**	**0.33**	**0.68**
6							**0.19**	**0.43**	**0.49**	**0.57**	**0.16**	**0.37**	**0.34**	**0.36**	**0.39**	**0.63**
7								**0.18**	**0.23**	**0.29**	**0.27**	0.14	**0.19**	**0.22**	**0.20**	**0.29**
8									**0.50**	**0.53**	**0.43**	**0.50**	**0.31**	**0.38**	**0.31**	**0.73**
9										**0.71**	**0.39**	**0.61**	**0.40**	**0.47**	**0.48**	**0.78**
10											**0.40**	**0.56**	**0.48**	**0.58**	**0.52**	**0.83**
11												**0.43**	**0.28**	**0.27**	**0.27**	**0.55**
12													**0.44**	**0.41**	**0.33**	**0.74**
13														**0.32**	**0.31**	**0.63**
14															**0.59**	**0.63**
15																**0.59**

Bold values are for P < 0.05.

### Reliability

Internal consistency of the pediatric QoR-15 assessed by Cronbach’s alpha was 0.90. Test–retest concordance correlation coefficient was 0.92 (95% CI [0.83–0.96]). Split-half alpha was 0.74.

### Responsiveness

Cohen Size effect and standardized response mean are reported in Table 3.

**Table 3:** Mean preoperative, postoperative, change and responsiveness of the pediatric Quality of Recovery French scale (QoR-15F) score.

Pediatric QoR-15F item	Pre-op pediatric QoR-15F	Post-op pediatric QoR-15F	Mean change	CI 95%	CI 95%	% change	Cohen’s effect size	Standardized response mean
Q1	9.5 (1.1)	9.4 (1.3)	− 0.1	− 0.3	0.2	− 1.0	− 0.1	− 0.1
Q2	8.9 (1.9)	7.5 (2.8)	− 1.4	− 1.8	− 0.9	− 15.4	− 0.7	− 0.5
Q3	7.9 (2.1)	7.0 (2.5)	− 0.9	− 1.4	− 0.5	− 11.8	− 0.5	− 0.3
Q4	8.6 (1.8)	6.6 (3.3)	− 2.0	− 2.6	− 1.5	− 23.6	− 1.1	− 0.6
Q5	9.6 (1.3)	6.5 (3.4)	− 3.1	− 3.7	− 2.5	− 32.1	− 2.3	− 0.8
Q6	9.0 (1.9)	8.5 (2.2)	− 0.5	− 0.9	− 0.1	− 5.6	− 0.3	− 0.2
Q7	9.4 (1.2)	9.5 (1.3)	0.1	− 0.2	0.3	0.7	0.1	0.1
Q8	8.3 (2.3)	4.3 (3.5)	− 3.9	− 4.5	− 3.4	− 47.7	− 1.7	− 1.1
Q9	8.6 (1.8)	6.6 (2.9)	− 2.0	− 2.4	− 1.5	− 23.1	− 1.1	− 0.7
Q10	9.1 (1.7)	7.5 (2.8)	− 1.6	− 2.0	− 1.1	− 17.3	− 0.9	− 0.6
Q11	8.0 (2.5)	6.3 (2.6)	− 1.7	− 2.3	− 1.1	− 21.1	− 0.7	− 0.5
Q12	8.8 (2.4)	6.5 (3.5)	− 2.3	− 2.9	− 1.7	− 26.0	− 0.94	− 0.6
Q13	9.7 (1.2)	7.9 (3.0)	− 1.8	− 2.3	− 1.3	− 18.5	− 1.6	− 0.6
Q14	7.6 (2.7)	8.3 (2.6)	0.6	0.1	1.1	8.0	0.2	0.2
Q15	9.1 (1.9)	8.7 (2.5)	− 0.4	− 0.8	0.04	− 4.4	− 0.2	− 0.2
Total	132.1 (14.1)	111.0 (27.0)	− 21.2	− 25.1	− 17.2	− 16.0	− 1.5	− 0.9

Mean (SD) or (95% confidence interval).

*Pre-op* preoperatively, *Post-op* postoperatively, *CI* confidence interval.

### Acceptability and feasibility

The completion rate for administered questionnaires was 100%. The mean times to complete the pediatric QoR-15F were 4.0 (1.9) and 4.3 (1.5) min in the preoperative and postoperative periods, respectively.

## Discussion

Our study shows that a pictorial adaptation of the Quality of Recovery 15 scale allows for the measurement of postoperative quality of recovery in children. The pictorial pediatric QoR-15 scale underwent the same psychometric validation process as the adult numerical versions^8,13^. Construct validity was confirmed by the convergence (i.e., statistical correlation) between the self-rating of each child’s global recovery and their postoperative QoR-15 score. The correlation coefficient between the postoperative pediatric QoR-15F total score and patient rated global recovery was 0.72, which is even stronger than was observed in the adult English (0.68)^8^ and French (0.60)^13^ validation studies.

Most the prior constructed validity hypotheses were confirmed. For example, the difference in the pediatric QoR-15 between the minor, intermediate and major surgery groups was significant. There was an excellent psychometric reliability, responsiveness, acceptability and feasibility of the pictorial pediatric QoR-15 scale.

There was no statistically significant difference between the group of patients with and without complications. This result might be explained by the very small number of early postoperative complications in our cohort, which is supported by a better global postoperative recovery in our pediatric cohort compared to the French adult validation study (postoperative QoR-15 111 (27) and 103 (21) in the French pediatric and adult studies, respectively).

We believe that pediatric surgical patients may benefit from the introduction of an adapted QoR-15 tool for several reasons. Monitoring quality of recovery is an important clinical tool for perioperative clinicians. For example, it allows for Enhanced Recovery After Surgery (ERAS) program implementation and monitoring. The QoR-15 is also a reliable outcome assessment tool for surgical patients^5^, and therefore is an additional tool that can be used for the assessment of postoperative recovery. Postoperative outcomes such as mortality and prolonged hospital length of stay are interesting but limited endpoints. For example, postoperative mortality in children undergoing surgery is rare in the modern perioperative era and therefore has limited utility to measuring quality of care and the patient’s experience. In contrast, PROMS like the QoR-15 allows for assessment of the patient’s perspective. In addition, PROMS, like the QoR-15, can also be important tools for perioperative research^2^. As PROMS measure what is important to patients, i.e. perceived health status, they are meaningful study endpoints^20,21^. Several expert guidelines recommend the use of PROMS like QoR-15, for perioperative clinical research^2,5^. The minimal difference in the QoR-15 score that constitutes a meaningful change in health status has been described and is thus useful for researchers^22,23^.

Measuring perioperative patterns with PROMS like QoR-15 should enable clinicians to better understand the trajectory of a normal postoperative recovery. These normal recovery patterns, based on the patient perspective, can help clinicians to set more realistic expectations and goals for patients, and their caregivers, after surgery.

Our study has several limitations. First, we only included children aged 7 to 17 years old, accordingly to previous studies in the field of self-reported pediatric symptoms^17,19^, so the performance of this scale for younger pediatric patients may be different. For example, the concepts of “personal toilet” or “feeling depressed” may not have the same signification for the youngest patients. In addition, for the youngest patients, especially younger than 7 years old, quantification of symptoms may be challenging and heterogeneous.

Second, the study only included French speaking patients and family members so validation in different language settings will be required.

Our study has several strengths, including different type of surgeries, increasing the external validity of our study setup. We also applied a broadly accepted study design for psychometrical validation of this PROMS instrument.

## Conclusion

Our study shows the design and validity of a pictorial QoR-15 scale in a French speaking pediatric patient and family setting. This tool was shown to have validity, reliability, responsiveness and feasibility. This pictorial scale should be considered to monitor the perioperative health status of 7 to 17 years old pediatric patients undergoing surgery, e.g. as part of ERAS pathway, and as a tool in clinical research.

### Supplementary Information

Supplementary Figure 1.

## Data Availability

The datasets used and/or analyzed during the current study available from the corresponding author on reasonable request.
