# Supplementary material for: Pictorial adaptation of the quality of recovery 15 scale and psychometric validation into a pediatric surgical population
Source: Sci Rep. 2023 Aug 28;13:14085. doi: 10.1038/s41598-023-40673-w (PMC10462607; doi:10.1038/s41598-023-40673-w)
Supplement: Supplementary file 1 — Supplementary Figure 1. [file 41598_2023_40673_MOESM1_ESM.pdf]

Questionnaire QoR-15

(traduit et adapté d'après Stark et col.[1])

Date : _ _ / _ _ / _ _ Heure : _ _ h _ _ **Etude : RNI XXX – HUS N°XXX**

Préopératoire ☐ Postopératoire ☐

PARTIE A

Comment t'es-tu senti(e) durant les 24 dernières heures ?

1. capable de respirer facilement

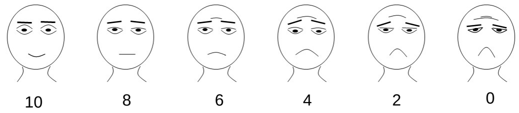

2. capable d'apprécier les aliments

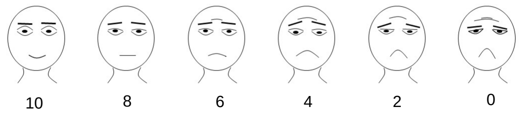

3. senti(e) reposé(e)

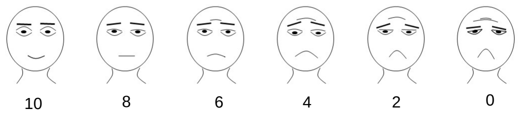

4. avoir bien dormi

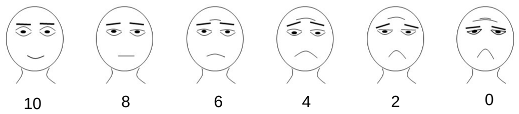

5. capable de faire sans aide ta toilette et tes soins d'hygiène

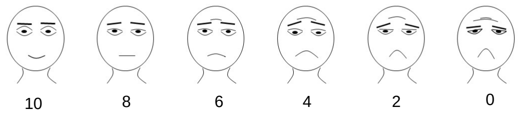

6. d'être capable de communiquer avec ta famille et tes amis

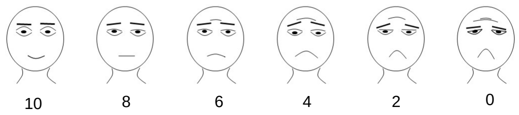

7. obtenir du soutien de la part des médecins et infirmières de l'hôpital

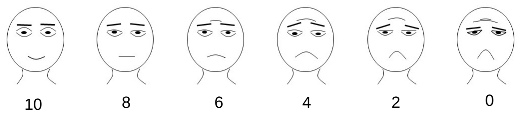

8. capable de retourner à l'école ou de reprendre tes activités habituelles à la maison

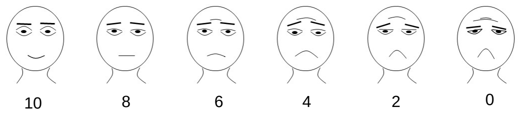

9. se sentir confortable et maître de la situation

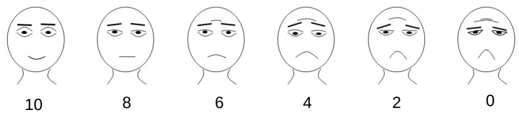

10. avoir une sensation générale de bien-être

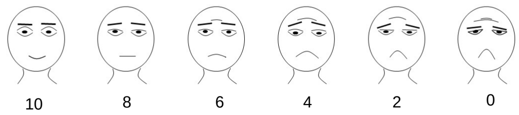

PARTIE B

As-tu éprouvé l'un des éléments suivants durant les dernières 24 heures ?

11. douleur modérée

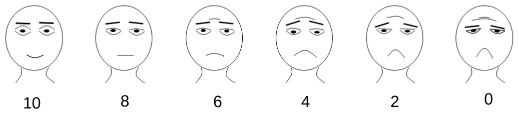

12. douleur sévère

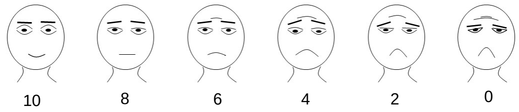

13. nausées ou vomissements

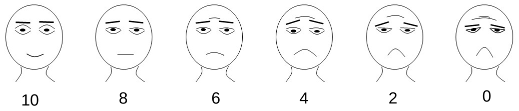

14. sensation d'inquiétude ou d'angoisse

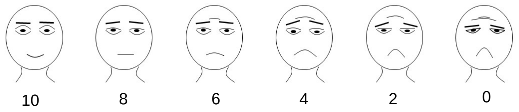

15. sentiment de tristesse ou de dépression

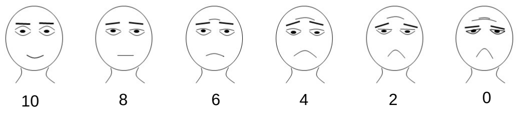

1.
Stark PA, Myles PS, Burke JA: Development and psychometric evaluation of a postoperative quality of recovery score: the QoR-15. Anesthesiology 2013; 118:1332–40
